# The Influence of Frailty on Total Hip Arthroplasty Outcomes: A Propensity-Matched Study of 90,660 Patients Using the Modified Frailty Index

**DOI:** 10.3390/jcm15072802

**Published:** 2026-04-07

**Authors:** Sri Tummala, Mehul M. Mittal, Hetsinhji Chavda, Tarun R. Sontam, Senthil N. Sambandam, Dane K. Wukich

**Affiliations:** 1 Department of Orthopaedic Surgery, University of Texas Southwestern Medical Center, 1801 Inwood Road, Dallas, TX 75390, USA; sr1@tamu.edu (S.T.); dane.wukich@utsouthwestern.edu (D.K.W.); 2 Department of Medical Education, Texas A&M School of Medicine, Baylor University Medical Center, 3500 Gaston Avenue, 6-Roberts, Dallas, TX 75246, USA; hetchavda@tamu.edu (H.C.); tarunsontam@tamu.edu (T.R.S.); 3 Department of Orthopaedics, University of Texas Southwestern Medical School, 5323 Harry Hines Blvd, Dallas, TX 75390, USA; mehul.mittal@utsouthwestern.edu; 4 Department of Prosthetics and Orthotics, University of Texas Southwestern Medical Center, Dallas, TX 75390, USA

**Keywords:** arthroplasty, replacement, hip, frailty, postoperative complications, risk assessment, propensity score, aged, comorbidity

## Abstract

**Background:** Frailty is a key predictor of adverse surgical outcomes in older adults. However, the prognostic utility of the 5-factor modified frailty index (mFI-5) for short- and long-term outcomes following total hip arthroplasty (THA) remains inadequately defined. This study assessed the association between frailty severity, measured by the mFI-5, and postoperative complications, implant survivorship, and mortality following primary THA in a large national cohort. **Methods:** This retrospective cohort study included 90,660 patients aged ≥50 years undergoing primary THA for osteoarthritis from 2003 to 2020 using the TriNetX research network. Patients were stratified by frailty severity based on mFI-5 scores: non-frail (0–1), moderately frail (2), and severely frail (≥3). Cases of fractures, polytrauma, or falls were excluded. Pairwise propensity score matching was adjusted for age, sex, race, and BMI. Outcomes included 90-day medical and surgical complications, healthcare utilization, and 2- and 5-year THA revision and mortality rates. Risk ratios (RRs) with 95% confidence intervals (CIs) and Bonferroni-corrected significance thresholds (*p* < 0.0167) were reported. **Results:** Severely frail patients had significantly increased risks of 90-day mortality (RR 4.41, 95% CI 2.22–8.74), acute kidney injury (RR 2.92), myocardial infarction (RR 3.61), and periprosthetic joint infection (RR 2.02) compared to non-frail patients. At five years, severely frail patients demonstrated a 58% higher revision risk (RR 1.58) and 23.0% mortality versus 6.9% in the non-frail cohort. A dose-dependent risk gradient was observed, with moderately frail patients exhibiting intermediate risks across all outcomes. **Conclusions:** Frailty severity, as measured by the mFI-5, was associated with a stepwise increase in short- and long-term complications and mortality following THA. The mFI-5 may serve as a practical, scalable tool for preoperative risk stratification, counseling, and resource planning in older adults undergoing primary THA.

## 1. Introduction

Total hip arthroplasty (THA) remains a cornerstone of modern orthopedic surgery, consistently demonstrating favorable long-term outcomes in patients with end-stage osteoarthritis, osteonecrosis, inflammatory arthropathies, and congenital hip disorders [1,2,3,4]. Nevertheless, the increasing prevalence of older patients undergoing THA presents distinct postoperative challenges, particularly heightened susceptibility to medical and surgical complications [5]. This evolving patient profile underscores the necessity of integrating validated perioperative risk stratification tools to optimize patient outcomes in patients undergoing THA.

Frailty, a multidimensional syndrome marked by diminished physiological resilience and multisystem dysregulation has gained prominence as a pivotal predictor of surgical outcomes in geriatric populations [6,7,8,9]. Recent evidence has found that frail individuals face disproportionately elevated risks of postoperative morbidity, including prolonged functional dependence, accelerated loss of independence in activities of daily living (ADLs), and increased mortality, even when adjusting for baseline comorbidities [10,11,12]. Although population-based analyses leveraging administrative datasets have consistently associated frailty with adverse events, prolonged hospitalization, escalated healthcare costs, and higher mortality, clinical adoption of frailty assessment remains limited by the absence of a standardized operational definition [11,13,14].

To address this knowledge–practice gap, the orthopedic literature has seen a proliferation of risk prediction models, including the Charlson Comorbidity Index, Elixhauser Comorbidity Method, and modified frailty indices (mFIs) [15,16,17]. Among these, the Canadian Study of Health and Aging Frailty Index, was a metric initially designed to quantify cumulative physiological deficits and has been validated across surgical populations for its robust association with adverse outcomes, including morbidity and mortality [18,19]. Subsequently, the 11-factor modified frailty index (mFI-11), derived from the American College of Surgeons National Surgical Quality Improvement Program (NSQIP) database, gained traction as a validated predictor of postoperative complications and mortality in diverse surgical cohorts [20,21,22]. However, the progressive exclusion of six variables from NSQIP documentation since 2012 necessitated the development of the five-factor variant (mFI-5), which retains clinical relevance while prioritizing pragmatic applicability [23]. The mFI-5 incorporates five variables routinely captured in clinical assessments including hypertension, chronic obstructive pulmonary disease, congestive heart failure, diabetes mellitus, and non-independent functional status.

The mFI-5 has demonstrated preserved prognostic accuracy alongside improved clinical practicality, relying exclusively on routinely collected clinical parameters [24]. Frailty severity is stratified via a cumulative scoring system (0–5), with higher scores correlating strongly with complications, prolonged hospitalization, mortality, and escalated healthcare expenditures [24]. This streamlined approach facilitates risk stratification, enabling clinicians to prioritize preoperative optimization in high-risk cohorts while supporting institutional resource stewardship through targeted interventions [25].

With over 450,000 THA procedures performed annually in the United States, a figure projected to increase steadily, investigating the independent prognostic role of frailty in this population remains timely and clinically relevant [26,27]. Furthermore, THA has been shown to effectively reduce the incidence of hip fractures by removing the operated hip from fracture risk, with recent population-level data demonstrating an approximately 10% reduction in age-standardized hip fracture incidence attributable to the increased prevalence of hip prostheses [28]. The purpose of this study was to assess the validity of the mFI-5 in THA patients and characterize postoperative complication profiles among frail patients undergoing THA, with the aim of enhancing preoperative risk stratification and informing patient counseling strategies tailored to frailty severity.

## 2. Methods

### 2.1. Data Source

This study utilized data from the TriNetX Research Network (https://trinetx.com, Cambridge, MA, USA), a comprehensive repository of healthcare data covering the United States, Canada, and Western Europe. The TriNetX network consolidates data from inpatient, outpatient, and emergency department visits provided by 112 healthcare organizations (HCOs) from the United States, encompassing more than 152 million unique patient records. This patient dataset is further enhanced by additional information sourced from over 100 commercial and government payers, including Medicare. This study was exempt from IRB approval since the data were de-identified and publicly available.

### 2.2. Study Design

**Figure 1:**
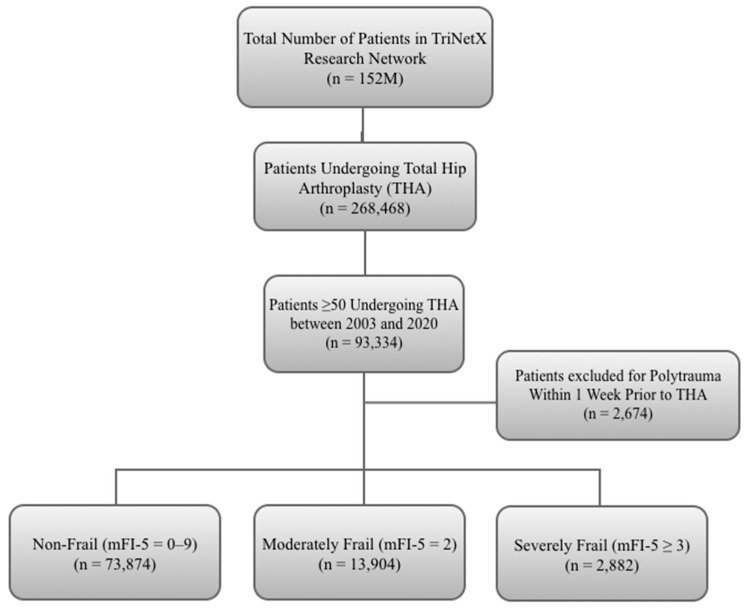
STROBE Patient Selection Flowchart.

Patients ≥ 50 years who underwent primary THA for osteoarthritis, identified by the Current Procedural Terminology (CPT) code 27130, between the years 2003 and 2020, were included in the analysis. Patients were subsequently categorized into three cohorts based on their frailty level using the mFI-5: (1) non-frail patients with zero or one documented comorbidity from the mFI-5 criteria; (2) moderately frail patients with exactly two documented mFI-5 comorbidities; and (3) severely frail patients with three or more documented mFI-5 comorbidities. Diagnoses pertaining to the five mFI-5 criteria were required to be documented within one year preceding the THA procedure, ensuring accurate representation of frailty status at the time of surgery. The age threshold of ≥50 years was selected based on established literature demonstrating that frailty prevalence and its clinical impact on surgical outcomes become most pronounced in this population, and because the vast majority of elective primary THA procedures are performed in patients aged 50 years and older, consistent with prior large-scale arthroplasty database studies [15,23]. To maintain the homogeneity of elective procedures and exclude acute trauma-related interventions, patients with a diagnosis of polytrauma, traumatic falls, and hip fractures (ICD-10: T07, W00–W19, S72.0–S72.2) recorded within one week preceding the THA were excluded. Additionally, patients who underwent revision THA, identified by CPT codes 27134 (acetabular and femoral component revision), 27137 (acetabular component revision), and 27138 (femoral component revision), were excluded to ensure the study population was limited to primary THA procedures. The one-week exclusion window for trauma diagnoses was specifically chosen to capture acute polytrauma and fall-related cases presenting for urgent or emergent THA, while preserving patients with remote trauma histories who subsequently underwent elective THA for osteoarthritis. This approach is consistent with established methodologies in large database studies that aim to distinguish elective from non-elective arthroplasty procedures [6,29]. All patient selection and cohort stratification utilized validated CPT, ICD-9, and ICD-10 coding methodologies. This retrospective cohort study adhered to the Strengthening the reporting of observational studies in epidemiology (STROBE) guidelines for observational research, with detailed patient selection criteria illustrated in Figure 1.

### 2.3. Outcomes Evaluated

The outcomes evaluated included short-term and long-term postoperative complications and healthcare utilization. At 90 days postoperatively, we evaluated major medical complications (mortality, superficial and deep surgical site infections (SSI), wound dehiscence, transfusion, myocardial infarction (MI), pulmonary embolism (PE), deep vein thrombosis (DVT), acute kidney failure (AKF), hematoma, pneumonia, anemia, sepsis, and tripping/slipping/falls (STF)), healthcare utilization outcomes (inpatient readmissions (IR) and emergency department (ED) visits), and surgical complications (THA revision, periprosthetic fracture (PPF), periprosthetic dislocation (PPD), periprosthetic mechanical complication (PMC), periprosthetic joint infection (PJI), aseptic loosening (ASL), and proximal femur fracture (PFF)). The same surgical complications along with mortality were also assessed at 2-year and 5-year time points.

### 2.4. Propensity Score Matching

To minimize confounding and ensure comparability between the non-frail, moderately frail, and severely frail patient cohorts, pairwise 1:1 propensity score matching was employed for all compared cohorts. Matching was performed using a greedy nearest-neighbor algorithm (caliper width: 0.1 pooled SDs), with key matching variables including age, sex, race, and categorized body mass index. These variables were carefully selected based on their demonstrated association with postoperative complications in an initial regression analysis. Covariate balance between matched cohorts was quantified using standardized mean differences (SMD), with post-matching SMD < 0.1 indicating adequate balance.

### 2.5. Statistical Tools and Data Analyses

Risk Ratio (RR), supplemented by absolute risk measures, was utilized to quantify differences in complication rates between the three patient cohorts. Risk ratios were presented alongside 95% confidence intervals (CIs) to provide precise estimates of risk differences. Statistical significance of categorical variables was assessed using Chi-square and Fisher’s exact tests, while continuous variables were analyzed with Student’s *t*-test.

Given our analysis involved multiple pairwise comparisons among the three frailty cohorts (non-frail vs. moderately frail, non-frail vs. severely frail, and moderately frail vs. severely frail), a Bonferroni correction was applied for all tests to adjust for the increased risk of type I error resulting from conducting multiple statistical tests. The Bonferroni method was specifically chosen as it provides a conservative correction by dividing the standard alpha level (0.05) by the number of comparisons (n = 3). Thus, statistical significance was defined as a *p*-value of <0.0167 (0.05/3), ensuring control over the family-wise error rate. All statistical analyses were performed using R (R Statistical Software version 4.5.0, Vienna, Austria) and within TriNetX.

## 3. Results

### 3.1. Analysis of Medical Outcomes at 90-Day Following THA

Moderately frail patients demonstrated significantly elevated risks of mortality, transfusion, MI, PE, DVT, AKF, pneumonia, anemia, sepsis, and STF compared to non-frail patients (*p* < 0.0167; Table 1). Severely frail patients exhibited further risk escalation, with higher rates of mortality, transfusion, MI, AKF, pneumonia, anemia, sepsis, and STF relative to non-frail individuals (Table 2). Direct comparison between frailty cohorts revealed that severe frailty independently was associated with increased risks of mortality, AKF, pneumonia, and anemia over moderate frailty (Table 3).

### 3.2. Analysis of Surgical Outcomes at 2-Years Following THA

At two years following THA, moderately frail patients exhibited significantly increased risks of THA revision, PPD, PMC, PJI, and mortality compared to non-frail patients (Table 4). Severely frail patients demonstrated further risk escalation, with higher rates of THA revision, PPF, PPD, PJI, and mortality relative to non-frail counterparts (Table 5). Direct comparison between frailty cohorts revealed that severe frailty conferred incremental risks of PPD, PJI, and mortality over moderate frailty (Table 6).

### 3.3. Analysis of Surgical Outcomes at 5-Years Following THA

At five years following THA, moderately frail patients had significantly increased risks of THA revision, PPF, PPD, PMC, PJI, and mortality compared to non-frail patients (Table 4). Severely frail patients, when compared to non-frail patients, exhibited significantly higher risks of THA revision, periprosthetic fracture (PPF), PPD, PJI, proximal femur fracture (PFF), and mortality compared to non-frail patients (Table 5). Direct comparison between frailty cohorts revealed that severe frailty was associated with higher risks of THA revision, periprosthetic fracture (PPF), PPD, PJI, proximal femur fracture (PFF), and mortality compared to moderately frail patients at 5 years (Table 6).

## 4. Discussion

This study found frailty, as measured by mFI-5, to be associated with an increased risk for adverse postoperative outcomes, revealing increases in both medical and surgical complication rates across non-frail, moderately frail, and severely frail patient cohorts. Specifically, frail patients demonstrated a significantly elevated 90-day risk of all-cause mortality, acute cardiopulmonary, hematologic, and thromboembolic complications. Concurrently, frailty status was linked to diminished mid-term implant survivorship, as evidenced by higher mechanical complications and THA revision rates at 2- and 5-year postoperative intervals. Furthermore, frailty correlated with heightened 90-day postoperative healthcare utilization, marked by increased frequencies of unplanned readmissions and emergency department encounters. These findings collectively highlight frailty as a potential determinant of both short-term postoperative morbidity/mortality and longitudinal implant failure following THA and underscores the mFI-5’s pragmatic clinical utility in preoperative risk assessment to aid surgeons in patient counseling and targeted optimization protocols to mitigate perioperative vulnerability.

An important consideration in interpreting these findings is the relationship between age and frailty. Frailty prevalence increases with advancing age, and the mFI-5 components, hypertension, COPD, congestive heart failure, diabetes mellitus, and functional dependence, are themselves age-associated conditions. To address this potential confounding, propensity score matching was performed on age, sex, race, and BMI, thereby balancing age distributions across frailty cohorts and isolating the independent contribution of frailty beyond demographic factors. Nevertheless, the observed dose-dependent relationship between frailty severity and adverse outcomes, even after age-matching, supports the conceptualization of frailty as a distinct biological construct that captures cumulative physiological vulnerability beyond chronological age alone.

**Figure 2:**
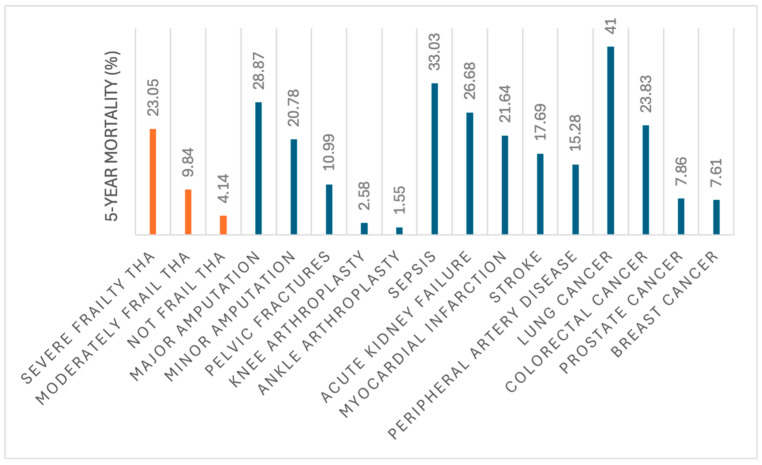
Mortality Risk at Five Years Among Frail THA Patients and Patients with Common Orthopedic Conditions, Systemic Complications, and Cancers.

A pronounced dose-dependent relationship between frailty and mortality was observed, as severely frail patients incurred a 4.4-fold increased risk of 90-day mortality compared to non-frail counterparts (RR: 4.409, 95% CI: 2.223–8.744), while moderately frail patients demonstrated a 2.8-fold elevated risk (RR: 2.75, 95% CI: 1.555–4.862). These findings corroborate large-scale meta-analyses encompassing over 460,000 arthroplasty patients, which identified frailty as an independent predictor of a threefold mortality risk following THA [29,30,31]. Mechanistically, the heightened postoperative vulnerability observed in frail patients may be attributable to multiple factors, including diminished physiologic reserve and compromised stress-response homeostasis, which may collectively predispose this population to adverse perioperative outcomes [30]. Notably, severely frail patients undergoing THA exhibited a substantially high five-year mortality rate of approximately 23%, underscoring a critical need for heightened clinical attention, especially when viewed in the context of five-year mortality rates of other major orthopedic procedures, medical conditions, and common cancers. Specifically, this mortality risk surpasses rates observed with pelvic fractures (11%), minor amputations (21%), knee arthroplasty (3%), ankle arthroplasty (2%), prostate cancer (8%), breast cancer (8%), myocardial infarction (22%), peripheral artery disease (15%), and stroke (18%) (Table 7 and Figure 2). This comparative analysis emphasizes the imperative for comprehensive preoperative counseling with patients and their families in the context of severe frailty, given the significant postoperative mortality risks identified.

Beyond mortality, frail patients demonstrated a significantly higher incidence of major cardiopulmonary complications. Severely frail individuals faced a substantially elevated risk of myocardial infarction (RR: 3.608, 95% CI: 1.777–7.324), with pneumonia risk exhibiting a progressive escalation commensurate with frailty severity (non-frail vs. severely frail: RR: 2.441, 95% CI: 1.390–4.286). These trends likely stem from the multifactorial pathophysiology of frailty, including impaired cardiopulmonary reserve, diminished respiratory muscle function, and compromised pulmonary clearance mechanisms, all of which amplify susceptibility to perioperative pulmonary compromise and ischemic cardiac events [30].

Analysis of major medical complications revealed a pattern of multisystem vulnerability associated with frailty, manifesting across renal, infectious, and hematologic systems. Severely frail patients demonstrated a 2.4-fold increased risk of sepsis (RR: 2.352, 95% CI: 1.369–4.042) and a 2.9-fold elevated risk of acute kidney injury (AKI; RR: 2.924, 95% CI: 1.895–4.513) within 90 days postoperatively compared to non-frail patients. These findings align prior literature implicating frailty as a driver of multi-organ dysfunction rather than isolated complications [32,33]. This multisystem propensity is further corroborated by Zalikha et al. in a total joint arthroplasty cohort, where frailty conferred a 1.5-fold risk of periprosthetic joint infection (PJI; 95% CI: 1.250–1.861) paralleling our study’s findings [34]. The convergence of these outcomes across studies suggests that frailty-associated pathophysiologic mechanisms, marked by inflammatory dysregulation and impaired tissue repair, may heighten systemic susceptibility to surgical stress, amplifying perioperative risk [7].

Beyond multisystem organ dysfunction, frailty was also disproportionately associated with elevated rates of hematologic complications, including transfusion (RR range: 1.559–2.098) and postoperative anemia (RR range: 1.363–1.818), relative to non-frail patients within the 90-day postoperative period. These results align with a recent study that found a 1.8-fold risk of postoperative anemia (95% CI: 1.401–2.360) in frail arthroplasty patients [34]. These outcomes may stem from baseline hematologic compromise, such as preoperative anemia or diminished erythropoietic reserve, and reduced physiologic tolerance to intraoperative blood loss. Notably, a graded risk escalation was observed across frailty strata, with severely frail patients demonstrating a 1.26-fold higher transfusion risk (RR: 1.262, 95% CI: 0.965–1.649) and 1.52-fold greater anemia incidence (RR: 1.516, 95% CI: 1.177–1.953) compared to moderately frail counterparts.

The observed risk gradient in our cohort supports the concept of frailty as a continuum, with moderately frail patients showing intermediate rates of multiorgan complications such as acute kidney injury (RR: 1.579, 95% CI: 1.095–2.277) and pneumonia (RR: 2.015, 95% CI: 1.183–3.431) compared to non-frail individuals. These risks were further amplified in the severely frail group, as evidenced by a rise in 90-day mortality from 0.4% in non-frail patients to 1.6% in the severely frail cohort (RR: 4.409, 95% CI: 2.223–8.744). Severity-linked relationship aligns with prior work by Traven et al., who documented a 25% incremental increase in 30-day morbidity risk per mFI-5 point (RR: 1.25) among arthroplasty patients [15]. Our findings corroborate this pattern, demonstrating that progressive frailty increments are associated with heightened cardiac morbidity (myocardial infarction RR: 3.608, 95% CI: 1.777–7.324) and pulmonary complications (pneumonia RR: 2.441, 95% CI: 1.390–4.286).

Frailty’s clinical impact extended beyond perioperative medical morbidity to encompass incremental surgical complication risks, including PJI, PPD, and mechanical failure. At 90 days postoperatively, severely frail patients exhibited a 2.8% incidence of PJI (versus 1.4% in non-frail; RR: 2.022, 95% CI: 1.382–2.960) and a 2.2% rate of PPD (versus 1.0% in non-frail; RR: 2.229, 95% CI: 1.431–3.473). By 5-year follow-up, these disparities intensified, with PJI incidence rising to 5.3% in the severely frail cohort (versus 2.8% non-frail; RR: 1.893, 95% CI: 1.445–2.479) and PPD to 4.3% (versus 2.5% non-frail; RR: 1.740, 95% CI: 1.303–2.325). These trends align with prior evidence identifying frailty as a risk amplifier for biofilm-related infections and instability-driven arthroplasty complications [1,6,35]. Mechanistically, these associations may arise from frailty-related sarcopenia, as evidenced by elevated anemia rates and systemic inflammation, as reflected in heightened sepsis risk. Such pathophysiologic derangements are known to impair soft-tissue healing and osseointegration fostering an environment conducive to prosthetic micromotion, microbial colonization, and delayed implant failure [35,36]. 

The longitudinal implications of surgical complications were most critically contextualized by THA revision rates, which demonstrated a pronounced frailty-dependent gradient. Severely frail patients exhibited a 2.8% 90-day revision risk (versus 1.7% in non-frail; RR: 1.629, 95% CI: 1.141–2.325), escalating to 6.4% at 5 years (versus 4.0% non-frail; RR: 1.579, 95% CI: 1.255–1.987). A dose-dependent relationship was evident across frailty strata, with severe frailty conferring a 60% greater revision risk relative to moderate frailty at both 2- and 5-year intervals (RR: 1.6, 95% CI: 1.3–2.0). This trajectory likely originates from early postoperative complications such as PJI and PPD, which disproportionately burdened frail patients within 90 days. As posited in prior studies, these acute complications may precipitate cumulative biomechanical stress, culminating in late aseptic loosening (5-year RR: 1.357, 95% CI: 1.010–1.823) and elevated 5-year mortality (23.0% vs. 6.9% non-frail; RR: 3.34, 95% CI: 2.871–3.886) [6,29].

Notably, a statistically significant association between severe frailty and PFF was observed at the 5-year follow-up. Severely frail patients demonstrated a 2.7-fold increased risk of PFF compared to non-frail counterparts (RR: 2.692, 95% CI: 1.491–4.863), and a nearly two-fold elevated risk relative to moderately frail patients (RR: 1.915, 95% CI: 1.132–3.239). Notably, this risk disparity was absent in earlier postoperative intervals, suggesting a delayed manifestation of skeletal fragility that aligns with progressive frailty trajectories. Given the cohort’s age threshold (≥50 years), this temporal pattern may reflect the cumulative burden of osteoporotic remodeling and sarcopenia-driven bone density depletion, pathophysiologic processes that exacerbate skeletal vulnerability to low-energy fractures over time [35,36].

In parallel with these clinical outcomes, frailty was also associated with heightened early postoperative healthcare utilization, with severely frail patients exhibiting elevated 90-day IR (2.9% vs. 1.8% non-frail; RR: 1.615, 95% CI: 1.126–2.317) and ED visit rates (6.6% vs. 4.4%; RR: 1.509, 1.150–1.979). Moderately frail patients showed intermediate utilization (IR: 3.1% vs. 2.5%, RR: 1.344; ED: 6.0% vs. 4.7%, RR: 1.230), aligning with Tram et al.’s who reported a 1.7-fold increase in readmissions (95% CI: 1.4–2.1) and 40% higher hospital costs among frail THA patients, despite their 30-day focus [35]. This utilization likely reflects two pathways: direct sequelae of frailty-driven complications such as sepsis (RR: 2.352) and acute kidney injury (RR: 2.924), and (2) instability-related ED presentations (e.g., PPD [RR: 2.229] or anemia [RR: 1.818]). Additionally, the mFI-5 variables, including functional dependence and cardiometabolic disease, may also play a role in exacerbating recovery barriers, prolonging reliance on acute care resources.

From a clinical perspective, the present findings raise important considerations regarding the borders of qualification for THA in frail patients. While severe frailty was associated with markedly elevated complication and mortality rates, it is critical to emphasize that frailty should not serve as an absolute contraindication to THA. Rather, these data may inform a more nuanced, shared decision-making framework in which the anticipated benefits of pain relief, functional restoration, and improved quality of life are carefully weighed against the individualized perioperative risk profile. For patients with moderate to severe frailty, preoperative optimization strategies, including prehabilitation programs targeting nutritional status, cardiopulmonary fitness, and functional capacity, may serve to attenuate perioperative risk and improve postoperative trajectories. Furthermore, enhanced postoperative monitoring protocols, including extended rehabilitation and closer surveillance for early complications such as PJI and PPD, may be warranted in this population to mitigate the observed frailty-driven risk gradient.

Clinically, the mFI-5 may offer a practical framework for identifying patients at elevated risk of complications following THA. Its simplicity, reliance on commonly documented clinical variables, and applicability to large datasets make it a potentially useful adjunct to existing preoperative assessment tools. While other frailty indices such as the Charlson Comorbidity Index, HEMA score, or Clinical Frailty Scale are also used in surgical populations, the comparative performance of these tools in arthroplasty-specific cohorts remains an area for further investigation. Incorporating frailty assessment into routine workflows could help inform discussions around anticipated recovery, postoperative support needs, or the potential utility of prehabilitation strategies, particularly for patients with moderate to severe frailty. Rather than serving as a barrier to surgery, frailty stratification may support more individualized care planning and shared decision-making. Ongoing comparison of frailty indices in orthopedic cohorts will be useful to identify which tools offer the optimal balance of prognostic accuracy and clinical feasibility.

This study has several limitations that should be considered. The retrospective observational design of this study using a large database did not allow us to account for unmeasured confounding variables such as specific nuances of surgical technique or implant choice could have influenced outcomes alongside frailty. While propensity score matching and multivariable adjustments were employed to mitigate selection bias and balance baseline characteristics, residual confounding may persist due to the non-randomized allocation of frailty severity. Furthermore, administrative datasets reliant on ICD-10-CM and CPT coding may also lead to under captured complications managed in non-affiliated facilities or undocumented revision procedures performed at external institutions, introducing potential outcome misclassification. Nevertheless, the consistency of our findings with prior frailty–arthroplasty literature [4,14,30], and the utilization of propensity-matched cohorts strengthen the internal validity of our findings. However, future prospective studies incorporating granular surgical variables, patient-reported outcomes, and implant survivorship registries will be critical to corroborate these associations.

## 5. Conclusions

This comprehensive matched-cohort analysis found that increasing frailty severity, quantified by the mFI-5, exhibits an independent, stepwise association with elevated risks of adverse outcomes following THA. Patients with moderate frailty demonstrated significantly increased surgical risks, including a 2.1-fold adjusted risk of major cardiopulmonary complications and a 60% increased likelihood of revision surgery at five years, compared to non-frail patients. Severely frail patients experienced substantially greater morbidity and mortality, manifesting as a 4.3-fold increased risk for all-cause mortality and a cumulative 5-year revision incidence of 6.4%. Notably, five-year mortality rates reached 23% in the severely frail cohort and 10% in the moderately frail cohort, rates exceeding those reported for several major orthopedic conditions and selected malignancies. These findings underscore the mFI-5 as a robust, clinically relevant prognostic tool for surgical risk stratification in THA candidates. Orthopedic surgeons may integrate the mFI-5 into preoperative assessment as it may provide a validated, data-driven approach to identify high-risk patients who may benefit from enhanced preoperative counseling and risk-stratified postoperative care pathways to mitigate complication risks following THA.

## Figures and Tables

**Table 1:** Outcomes of Moderately Frail vs. Not Frail at 90-Days—Matched.

Measure	Moderately Frail n (% of Cohort)	Not Frail n (% of Cohort)	Risk Ratio	95% CI	*p*-Value
Medical Complications					
Mortality	76 (0.56%)	45 (0.33%)	1.691	(1.170, 2.443)	<0.0167
Superficial Surgical Site Infection	16 (0.12%)	18 (0.13%)	0.889	(0.454, 1.742)	0.73
Deep Surgical Site Infection	<10	<10	-	-	-
Wound Dehiscence	133 (0.99%)	107 (0.79%)	1.25	(0.970, 1.611)	0.08
Transfusion	399 (3.07%)	262 (1.97%)	1.559	(1.337, 1.819)	<0.0167
Myocardial Infarction	64 (0.50%)	32 (0.24%)	2.066	(1.353, 3.156)	<0.0167
Pulmonary Embolism	80 (0.61%)	50 (0.37%)	1.616	(1.136, 2.299)	<0.0167
Deep Vein Thrombosis	193 (1.48%)	146 (1.10%)	1.341	(1.083, 1.660)	<0.0167
Acute Kidney Failure	225 (1.85%)	118 (0.90%)	2.047	(1.640, 2.554)	<0.0167
Hematoma	17 (0.12%)	11 (0.08%)	1.546	(0.725, 3.300)	0.26
Pneumonia	131 (1.06%)	71 (0.55%)	1.942	(1.456, 2.589)	<0.0167
Anemia	497 (4.78%)	393 (3.51%)	1.363	(1.198, 1.552)	<0.0167
Sepsis	107 (0.81%)	55 (0.41%)	1.982	(1.433, 2.741)	<0.0167
Tripping, Slipping, and Falls	189 (1.57%)	109 (0.86%)	1.816	(1.437, 2.296)	<0.0167
Healthcare Utilization					
Inpatient Readmissions	380 (3.08%)	252 (1.96%)	1.575	(1.345, 1.843)	<0.0167
Emergency Department Visits	536 (5.78%)	494 (4.70%)	1.23	(1.092, 1.386)	<0.0167
Surgical Complications					
THA Revision	283 (2.10%)	211 (1.56%)	1.344	(1.126, 1.604)	<0.0167
Periprosthetic Fracture	110 (0.82%)	80 (0.59%)	1.381	(1.036, 1.840)	0.03
Periprosthetic Dislocation	180 (1.33%)	121 (0.89%)	1.494	(1.188, 1.878)	<0.0167
Periprosthetic Mechanical Complication	115 (0.87%)	86 (0.64%)	1.344	(1.018, 1.776)	0.04
Periprosthetic Joint Infection	241 (1.79%)	159 (1.17%)	1.525	(1.250, 1.861)	<0.0167
Proximal Femur Fracture	41 (0.30%)	28 (0.21%)	1.468	(0.909, 2.373)	0.12
Aseptic Loosening	16 (0.12%)	13 (0.10%)	1.232	(0.593, 2.560)	0.58

**Table 2:** Outcomes of Severely Frail vs. Not Frail at 90-Days—Matched.

Measure	Severely Frail n (% of Cohort)	Not Frail n (% of Cohort)	Risk Ratio	95% CI	*p*-Value
Medical Complications					
Mortality	44 (1.6%)	11 (0.4%)	4.409	(2.223, 8.744)	<0.0167
Superficial Surgical Site Infection	11 (0.40%)	<10	-	-	-
Deep Surgical Site Infection	<10	<10	-	-	-
Wound Dehiscence	41 (1.50%)	26 (0.90%)	1.588	(0.974, 2.589)	0.06
Transfusion	112 (4.40%)	57 (2.10%)	2.098	(1.532, 2.873)	<0.0167
Myocardial Infarction	32 (1.30%)	<10	3.608	(1.777, 7.324)	<0.0167
Pulmonary Embolism	23 (0.90%)	11 (0.40%)	2.144	(1.047, 4.390)	0.03
Deep Vein Thrombosis	34 (1.30%)	37 (1.40%)	0.952	(0.600, 1.512)	0.83
Acute Kidney Failure	65 (3.20%)	29 (1.10%)	2.924	(1.895, 4.513)	<0.0167
Hematoma	<10	<10	-	-	-
Pneumonia	36 (1.60%)	18 (0.70%)	2.441	(1.390, 4.286)	<0.0167
Anemia	132 (7.10%)	90 (3.90%)	1.818	(1.401, 2.360)	<0.0167
Sepsis	41 (1.60%)	19 (0.70%)	2.352	(1.369, 4.042)	<0.0167
Tripping, Slipping, and Falls	56 (2.40%)	20 (0.80%)	3.166	(1.906, 5.259)	<0.0167
Healthcare Utilization					
Inpatient Readmissions	72 (2.90%)	48 (1.80%)	1.615	(1.126, 2.317)	<0.0167
Emergency Department Visits	105 (6.60%)	93 (4.40%)	1.509	(1.150, 1.979)	<0.0167
Surgical Complications					
THA Revision	78 (2.80%)	48 (1.70%)	1.629	(1.141, 2.325)	<0.0167
Periprosthetic Fracture	33 (1.20%)	18 (0.60%)	1.859	(1.049, 3.294)	0.03
Periprosthetic Dislocation	62 (2.20%)	28 (1.00%)	2.229	(1.431, 3.473)	<0.0167
Periprosthetic Mechanical Complication	29 (1.10%)	20 (0.70%)	1.459	(0.827, 2.573)	0.19
Periprosthetic Joint Infection	78 (2.80%)	39 (1.40%)	2.022	(1.382, 2.960)	<0.0167
Proximal Femur Fracture	<10	<10	-	-	-
Aseptic Loosening	<10	<10	-	-	-

**Table 3:** Outcomes of Severely Frail vs. Moderately Frail at 90-Days—Matched.

Measure	Severely Frail n (% of Cohort)	Moderately Frail n (% of Cohort)	Risk Ratio	95% CI	*p*-Value
Medical Complications					
Mortality	44 (1.60%)	16 (0.60%)	2.75	(1.555, 4.862)	<0.0167
Superficial Surgical Site Infection	11 (0.40%)	<10	-	-	-
Deep Surgical Site Infection	<10	<10	-	-	-
Wound Dehiscence	41 (1.50%)	33 (1.20%)	1.243	(0.788, 1.960)	0.35
Transfusion	114 (4.40%)	94 (3.50%)	1.262	(0.965, 1.649)	0.09
Myocardial Infarction	32 (1.30%)	22 (0.80%)	1.597	(0.931, 2.741)	0.09
Pulmonary Embolism	23 (0.90%)	20 (0.70%)	1.177	(0.648, 2.138)	0.59
Deep Vein Thrombosis	35 (1.30%)	43 (1.60%)	0.827	(0.531, 1.288)	0.4
Acute Kidney Failure	65 (3.20%)	49 (2.00%)	1.579	(1.095, 2.277)	<0.0167
Hematoma	<10	<10	-	-	-
Pneumonia	37 (1.70%)	21 (0.80%)	2.015	(1.183, 3.431)	<0.0167
Anemia	132 (7.10%)	99 (4.70%)	1.516	(1.177, 1.953)	<0.0167
Sepsis	41 (1.60%)	26 (1.00%)	1.683	(1.033, 2.744)	0.03
Tripping, Slipping, Falls	56 (2.40%)	39 (1.60%)	1.531	(1.021, 2.295)	0.04
Healthcare Utilization					
Inpatient Readmissions	72 (2.90%)	81 (3.20%)	0.923	(0.675, 1.261)	0.61
Emergency Department Visits	106 (6.70%)	113 (6.00%)	1.11	(0.859, 1.434)	0.42
Surgical Complications					
THA Revision	79 (2.80%)	62 (2.20%)	1.275	(0.918, 1.770)	0.15
Periprosthetic Fracture	33 (1.20%)	29 (1.00%)	1.148	(0.699, 1.886)	0.58
Periprosthetic Dislocation	62 (2.20%)	38 (1.40%)	1.632	(1.093, 2.435)	<0.0167
Periprosthetic Mechanical Complication	29 (1.10%)	28 (1.00%)	1.036	(0.618, 1.737)	0.89
Periprosthetic Joint Infection	79 (2.80%)	50 (1.80%)	1.587	(1.118, 2.252)	<0.0167
Proximal Femur Fracture	<10	<10	-	-	-
Aseptic Loosening	<10	<10	-	-	-

**Table 4:** Moderately Frail vs. Not Frail Surgical Outcomes at 2 and 5 Years—Matched.

Measure	Moderately Frail n (% of Cohort)	Not Frail n (% of Cohort)	Risk Ratio	95% CI	*p*-Value
2 Year					
THA Revision	468 (3.50%)	337 (2.50%)	1.392	(1.212, 1.598)	<0.0167
Periprosthetic Fracture	189 (1.40%)	147 (1.10%)	1.291	(1.042, 1.599)	0.02
Periprosthetic Dislocation	291 (2.20%)	218 (1.60%)	1.34	(1.126, 1.594)	<0.0167
Periprosthetic Mechanical Complication	318 (2.40%)	244 (1.90%)	1.311	(1.112, 1.547)	<0.0167
Periprosthetic Joint Infection	390 (2.90%)	261 (2.00%)	1.504	(1.288, 1.756)	<0.0167
Proximal Femur Fracture	72 (0.50%)	51 (0.40%)	1.416	(0.990, 2.025)	0.06
Aseptic Loosening	60 (0.40%)	54 (0.40%)	1.113	(0.771, 1.606)	0.57
Mortality	481 (3.60%)	258 (1.90%)	1.866	(1.607, 2.167)	<0.0167
5 Year					
THA Revision	596 (4.40%)	484 (3.60%)	1.234	(1.097, 1.388)	<0.0167
Periprosthetic Fracture	292 (2.20%)	228 (1.70%)	1.286	(1.083, 1.527)	<0.0167
Periprosthetic Dislocation	370 (2.70%)	295 (2.20%)	1.259	(1.083, 1.465)	<0.0167
Periprosthetic Mechanical Complication	471 (3.50%)	368 (2.80%)	1.287	(1.125, 1.472)	<0.0167
Periprosthetic Joint Infection	500 (3.70%)	360 (2.70%)	1.398	(1.223, 1.597)	<0.0167
Proximal Femur Fracture	108 (0.80%)	78 (0.60%)	1.388	(1.039, 1.856)	0.03
Aseptic Loosening	103 (0.80%)	76 (0.60%)	1.357	(1.010, 1.823)	0.04
Mortality	1338 (9.80%)	764 (5.60%)	1.753	(1.610, 1.910)	<0.0167

**Table 5:** Severely Frail vs. Not Frail Surgical Outcomes at 2 and 5 Years—Matched.

Measure	Severely Frail n (% of Cohort)	Not Frail n (% of Cohort)	Risk Ratio	95% CI	*p*-Value
2 Year					
THA Revision	136 (4.90%)	81 (2.90%)	1.683	(1.285, 2.205)	<0.0167
Periprosthetic Fracture	65 (2.40%)	32 (1.10%)	2.06	(1.353, 3.135)	<0.0167
Periprosthetic Dislocation	94 (3.40%)	46 (1.60%)	2.057	(1.452, 2.916)	<0.0167
Periprosthetic Mechanical Complication	81 (2.90%)	62 (2.20%)	1.315	(0.948, 1.822)	0.1
Periprosthetic Joint Infection	117 (4.20%)	63 (2.20%)	1.878	(1.389, 2.539)	<0.0167
Proximal Femur Fracture	25 (0.90%)	11 (0.40%)	2.295	(1.131, 4.654)	0.02
Aseptic Loosening	11 (0.40%)	<10	-	-	-
Mortality	269 (9.50%)	62 (2.20%)	4.348	(3.315, 5.702)	<0.0167
5 Year					
THA Revision	178 (6.40%)	113 (4.00%)	1.579	(1.255, 1.987)	<0.0167
Periprosthetic Fracture	100 (3.60%)	51 (1.80%)	1.988	(1.425, 2.775)	<0.0167
Periprosthetic Dislocation	121 (4.30%)	70 (2.50%)	1.74	(1.303, 2.325)	<0.0167
Periprosthetic Mechanical Complication	130 (4.70%)	98 (3.50%)	1.335	(1.032, 1.726)	0.03
Periprosthetic Joint Infection	146 (5.30%)	78 (2.80%)	1.893	(1.445, 2.479)	<0.0167
Proximal Femur Fracture	40 (1.40%)	15 (0.50%)	2.692	(1.491, 4.863)	<0.0167
Aseptic Loosening	23 (0.80%)	20 (0.70%)	1.149	(0.633, 2.088)	0.65
Mortality	650 (23.00%)	195 (6.90%)	3.34	(2.871, 3.886)	<0.0167

**Table 6:** Severely Frail vs. Moderately Frail Surgical Outcomes at 2 and 5 Years—Matched.

Measure	Severely Frail n (% of Cohort)	Moderately Frail n (% of Cohort)	Risk Ratio	95% CI	*p*-Value
2 Year					
THA Revision	137 (4.90%)	108 (3.90%)	1.269	(0.992, 1.624)	0.06
Periprosthetic Fracture	65 (2.30%)	49 (1.80%)	1.339	(0.927, 1.932)	0.12
Periprosthetic Dislocation	94 (3.40%)	61 (2.20%)	1.541	(1.121, 2.118)	**<0.0167**
Periprosthetic Mechanical Complication	81 (2.90%)	74 (2.70%)	1.095	(0.803, 1.494)	0.57
Periprosthetic Joint Infection	118 (4.20%)	80 (2.90%)	1.481	(1.121, 1.958)	**<0.0167**
Proximal Femur Fracture	25 (0.90%)	14 (0.50%)	1.795	(0.935, 3.446)	0.07
Aseptic Loosening	11 (0.40%)	22 (0.80%)	0.499	(0.243, 1.028)	0.05
Mortality	268 (9.50%)	126 (4.50%)	2.127	(1.732, 2.611)	**<0.0167**
**5 Year**					
THA Revision	179 (6.40%)	134 (4.80%)	1.336	(1.075, 1.661)	**<0.0167**
Periprosthetic Fracture	100 (3.60%)	67 (2.40%)	1.506	(1.110, 2.043)	**<0.0167**
Periprosthetic Dislocation	121 (4.30%)	78 (2.80%)	1.551	(1.173, 2.052)	**<0.0167**
Periprosthetic Mechanical Complication	130 (4.70%)	99 (3.60%)	1.314	(1.017, 1.697)	0.04
Periprosthetic Joint Infection	147 (5.30%)	102 (3.70%)	1.447	(1.131, 1.853)	**<0.0167**
Proximal Femur Fracture	40 (1.40%)	21 (0.70%)	1.915	(1.132, 3.239)	**<0.0167**
Aseptic Loosening	23 (0.80%)	32 (1.10%)	0.718	(0.421, 1.223)	0.22
Mortality	650 (23.00%)	324 (11.50%)	2.006	(1.775, 2.268)	**<0.0167**

**Table 7:** Comparison of 5-Year Mortality Rates Among Frail THA Patients and Patients with Common Orthopedic Injuries, Systemic Complications, and Cancers.

Condition	Total Population (>50 yrs)	Mortality Within 5 Years n	5-Year Mortality (%)
Total Hip Arthroplasty (THA) Frailty			
Severe Frailty THA	2820	650	23.05%
Moderately Frail THA	13,603	1338	9.84%
Not Frail THA	69,216	2863	4.14%
Orthopedic Conditions/Procedures			
Major Amputation	37,750	10,900	28.87%
Minor Amputation	66,438	13,805	20.78%
Pelvic Fractures	141,824	15,592	10.99%
Knee Arthroplasty	344,146	8891	2.58%
Ankle Arthroplasty	5600	87	1.55%
Medical Conditions			
Sepsis	1,702,036	562,202	33.03%
Acute Kidney Failure	3,186,909	850,309	26.68%
Myocardial Infarction	1,579,227	341,796	21.64%
Stroke	1,749,134	309,343	17.69%
Peripheral Artery Disease	3,398,404	519,386	15.28%
Cancers			
Lung Cancer	72,228	29,613	41.00%
Colorectal Cancer	5595	1333	23.83%
Prostate Cancer	83,540	6569	7.86%
Breast Cancer	110,558	8415	7.61%

## Data Availability

The data that support the findings of this study are available from TriNetX. Restrictions apply to the availability of these data, which were used under license for this study. Data are available from https://trinetx.com with the permission from TriNetX.
